# High Resolution Detection and Analysis of CpG Dinucleotides Methylation Using MBD-Seq Technology

**DOI:** 10.1371/journal.pone.0022226

**Published:** 2011-07-11

**Authors:** Xun Lan, Christopher Adams, Mark Landers, Miroslav Dudas, Daniel Krissinger, George Marnellos, Russell Bonneville, Maoxiong Xu, Junbai Wang, Tim H.-M. Huang, Gavin Meredith, Victor X. Jin

**Affiliations:** 1 Department of Biomedical Informatics, The Ohio State University, Columbus, Ohio, United States of America; 2 Life Technologies, Carlsbad, California, United States of America; 3 Department of Pathology, The Norwegian Radium Hospital, Oslo University, Oslo, Norway; 4 Human Cancer Genetics Program, The Ohio State University, Columbus, Ohio, United States of America; National Institutes of Health, United States of America

## Abstract

Methyl-CpG binding domain protein sequencing (MBD-seq) is widely used to survey DNA methylation patterns. However, the optimal experimental parameters for MBD-seq remain unclear and the data analysis remains challenging. In this study, we generated high depth MBD-seq data in MCF-7 cell and developed a bi-asymmetric-Laplace model (BALM) to perform data analysis. We found that optimal efficiency of MBD-seq experiments was achieved by sequencing ∼100 million unique mapped tags from a combination of 500 mM and 1000 mM salt concentration elution in MCF-7 cells. Clonal bisulfite sequencing results showed that the methylation status of each CpG dinucleotides in the tested regions was accurately detected with high resolution using the proposed model. These results demonstrated the combination of MBD-seq and BALM could serve as a useful tool to investigate DNA methylome due to its low cost, high specificity, efficiency and resolution.

## Introduction

The advance of next generation sequencing technology has revolutionized the research field of transcriptional regulation and systems biology [1], [2], [3], [4]. ChIP-sequencing (ChIP-seq) has become a leading technology to interrogate *in vivo* protein-DNA interactions [5], [6], [7]. Recently, in addition to protein–DNA interactions, massively parallel sequencing has been used to identify open chromatin [8], histone modifications [5], [9], [10] and DNA methylation.

DNA methylation is one of the major epigenetic mechanisms that play an important role in a variety of cancers [11], [12], [13]. Several high throughput profiling techniques (MeDIP-seq [14], [15], [16], MIRA-seq [17], MBD-seq [18], MethylCap-seq [19], MethylC-seq [20], [21], BS-seq [22]) have been developed to study genome-wide methylation patterns. Affinity-based enrichment of methylated DNA sequences with methyl-CpG binding domain proteins followed by next generation sequencing (MBD-seq) [18] utilizing the MethylMiner™ Methylated DNA Enrichment kit has been shown to be a powerful alternative to MeDIP-seq and the whole methylome sequencing technology of BS-seq [18], [20], [21].

In MBD-seq experiments, high coverage of methylated CpG dinucleotides can be achieved by increased sequencing depth; however, as the sequencing depth increases so does the cost and the computational resource requirement. No optimal sequencing depth has been given by previous studies.

MACS [23], QuEST [24], SISSRs [25], PICS [26], and many other peak identification programs [27], [28], [29], [30], [31], [32] are developed for ChIP-seq data analysis; however, the majority of these programs were designed to locate transcription factor binding sites (TFBSs) from ChIP-seq data. DNA methylation sites differ from TFBSs in that methylated CpG dinucleotides are highly abundant in most differentiated cells thus the signal peaks in MBD-seq data are densely distributed. The characteristic of this type of data raises the demand for a computational analysis program with higher resolution, since the aforementioned programs fail to finely detect methylation level of CpG dinucleotides.

Several recent studies applied methods based on tags density [18], [33] or tags count normalized by the CpG density [34]. Similar to many of the peak detection programs [32], low resolution is the major disadvantage of using tags density based methods for MBD-seq data analysis.

In this study, we performed high sequencing depth MBD-seq in the human breast-cancer MCF-7 cell line. The result shows that with ∼100 million unique mapped tags (approximately five lanes using a GAII sequencer) from 500 mM and 1000 mM elutes the coverage of the MBD-seq data become close to a saturation point. A bi-asymmetric-Laplace model (BALM) was developed to analyze MBD-seq. We compared the resolution of BALM to that of several ChIP-seq analysis tools. The results demonstrate the program's superior ability to distinguish methylation statuses of closely positioned CpG sites.

This study demonstrates that MBD-seq combined with the new program is potentially a powerful tool to capture genome-wide DNA methylation profiles with high efficiency and resolution.

## Results

### Overview of experimental design and analysis

In MBD-seq experiments, fragments of methylated genomic DNA are precipitated by a specific capture protein. Single end of each of these fragments are sequenced simultaneously using high-throughput sequencing techniques. The precise locations of sequenced tags are recorded by mapping them to the reference genome.

**Figure 1:**
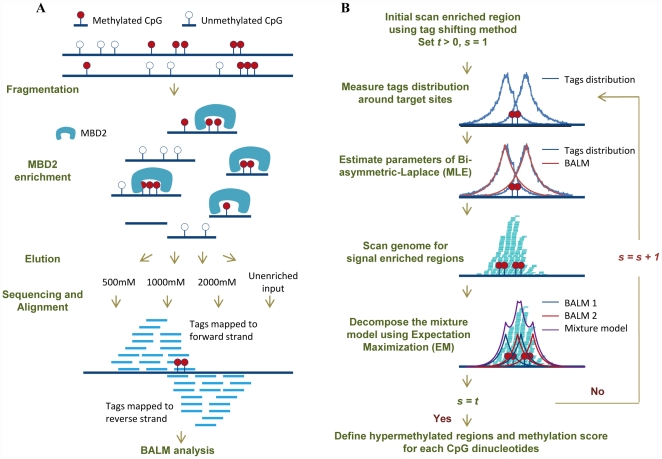
Overview of experimental design and analysis. A. Major steps of MBD-seq experiment. Methylated DNA was enriched by recombinant MBD2 protein. Different fractions of enriched DNA fragments were eluted under three different salt concentrations (500 mM, 1000 mM, 2000 mM). Together with unenriched fraction, four DNA fractions were then used to construct four standard fragment libraries. Libraries were sequenced using SOLiD™ 3 Analyzer and the resulting tags were aligned to the human genome using the Bowtie mapping software. B. BALM algorithm includes a step of initial scanning target sites using a tag shifting method; followed by modeling tag distribution around target sites as a BALM. Then scan the genome for enriched regions and predict target sites by maximizing the likelihood of the given tags within the enriched regions. BIC is used to determine the number of target sites.

In this study, a total number of ∼0.5 billion tags were generated from MBD-seq performed in MCF-7 cell line (**Table S1**). A flow chart of major steps of the experiments is shown in **Figure 1A**. Briefly, a recombinant form of the human MBD2 protein was applied to precipitate methylated DNA from genomic DNA. Three separate libraries was constructed under three different elution salt concentrations (500 mM, 1000 mM, 2000 mM) and were sequenced and subsequently aligned to Hg18 using the Bowtie mapping software [35] (****Materials and methods** MBD-seq section**).

**Figure 2:**
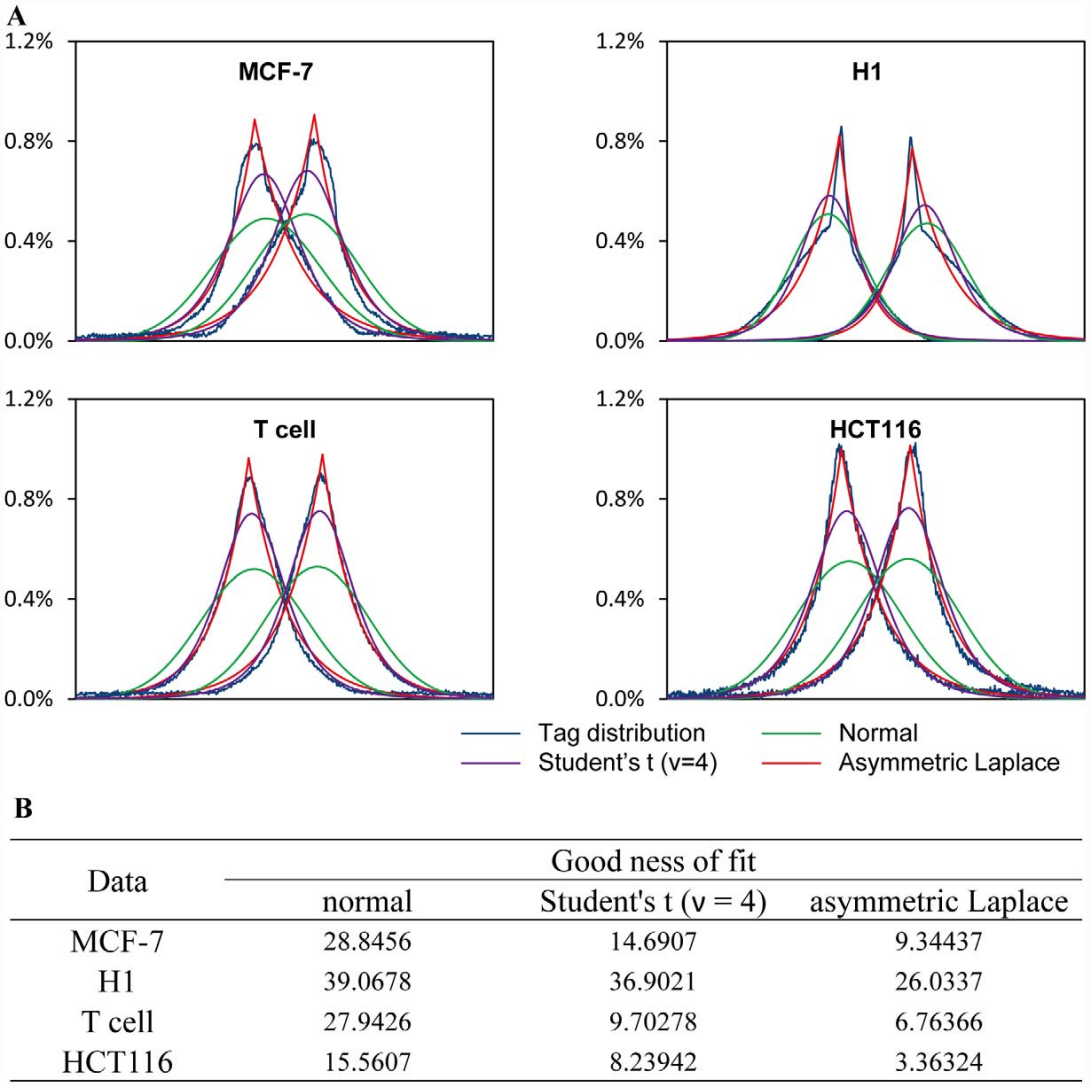
Bi-asymmetric-Laplace model. A. A plot of tags density surrounding the methylated CpG sites of MCF-7 MBD-seq and three public MBD-seq data in H1, T cell and HCT116 cell respectively (blue) and the fitted normal distribution (green), Student's t (ν = 4) distribution (purple) and bi-asymmetric-Laplace distribution (red). B. Asymmetric Laplace distribution gained smaller value of goodness of fit over the distribution of tags than normal distribution and t-distribution with 4 degree of freedom, indicates BALM captures the bimodal pattern more precisely.

To detect methylation level of each CpG dinucleotides, we present a statistic orientated algorithm, which is based on a bi-asymmetric-Laplace model. The model is aimed to precisely recapitulate the tags' bimodal distribution over target sites in a ChIP-seq experiment [23], [36] (**Figures 1A,B** **,** **2A**). This model is chosen based on the following facts. First, tags density decrease exponentially on both directions from the summit of each model. Second, an asymmetric exponential family distribution of the lengths of gel-electrophoresis-selected ChIP fragments is observed from paired-end sequencing data (**Figure S1**). More importantly, the proposed model bears a low value of goodness of fit in both MBD-seq and TFBSs ChIP-seq data compared to previously described Gaussian [24] and t-distribution model [26] (**Figure 2B** **, Figure S2**). A detailed list of estimated BALM parameters for each dataset is provided in **Table S2**. All four tested public transcription factor datasets have less than 10 million unique mapped tags, which demonstrates that obtaining an accurate model does not require extremely high sequencing depth (**Figure S2**). An overview of the algorithm is described below (**Figure 1B**):

1. Initial scan for enriched regions using a tag shifting method as in BELT [37]. If input data are available, a Fisher's exact test is performed to filter regions that are not significantly more enriched than the input and a genome region amplification index (*GRAI*) is calculated by the background enrichment level using the input data. Set *t*>0, *s* = 1 (*t* is the total number of iterations, *s* denotes the current iteration number).
2. Measure tag distribution over target sites.
3. Model the tag distribution over target sites as a bi-asymmetric-Laplace distribution and estimate the parameters using the maximum likelihood estimators [38], [39], [40].
4. Calculate weighted enrichment for each nucleotide in the genome. Weighted enrichment is defined as the tag enrichment weighted by tag's relative position to the nucleotide using the BALM. Then scan the genome for regions that have weighted enrichment higher than a local threshold which is weighted by the *GRAI*. Perform Fisher's exact test to filter regions that are not significantly enriched compared to input.
5. Within each enriched region, a BALM mixture is constructed. Center of each bi-asymmetric-Laplace distribution represent one target site. Unknown parameters are estimated using the EM algorithms [41], [42]. The number of components (each component can be interpreted as a target site) is determined by the Bayesian Information Criterion (BIC) [43], [44]. Then update the enriched regions and target sites list. For MBD-seq data, a methylation level is inferred from the mixture model for each CpG dinucleotides.
6. If *s<t*, increase *s* by 1, go to step 2, otherwise continue to step 7.
7. Output a list of enriched regions as well as the precise location of the predicted target sites occurring in these regions. For MBD-seq data, a file contains the methylation level of each CpG dinucleotides in the genome is generated.

The major novelty of the algorithm is that it accurately estimates each CpG dinucleotides' methylation level with high resolution in tag-enriched regions by maximizing the likelihood of given tags via expectation maximization (EM) [45], [46], [47]. A more extensive discussion of the algorithm is in the BALM algorithm section of **Materials and methods**.

### MBD-seq data analysis

**Figure 3:**
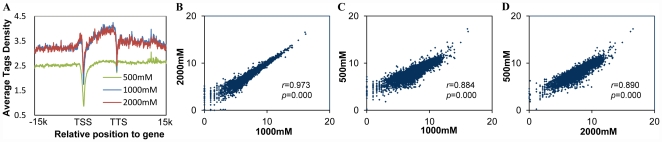
Correlation of tags density of different salt concentrations. A. A plot of average tags density *vs* a known gene (RefSeq HG18) for MBD-seq data in MCF-7 cell for three salt concentrations, 500 mM, 1000 mM and 2000 mM. The results showed a lower methylation levels occurs in the transcription start sites (TSS) and the transcription termination sites (TTS). Gene body and up down 15 kb region was plotted in this figure. The gene body region was evenly divided into 200 bins and 15 k bp up down gene body region was divided into 50 bp bins. Log tags count correlation analysis showed a higher correlation for 1000 mM *vs* 2000 mM (B, *r* = 0.973, *p* = 0.000) than 500 mM *vs* 1000 mM (C, *r* = 0.884, *p* = 0.000) and 500 mM *vs* 2000 mM (D, *r* = 0.890, *p* = 0.000).

The average tag density throughout known genes (RefSeq HG18) for all three experiments was plotted in **Figure 3A**. The results showed lower methylation levels occurs at both the transcription start sites (TSS) and the transcription termination sites (TTS), which is consistent with the observation in previous studies using bisulfite padlock probes with microarray [48] as well as bisulfite sequencing [49]. The tags distribution pattern of 1000 mM and 2000 mM elution are similar, however, 500 mM is substantially different from the other two (**Figure 3A**). Log tag count correlation analysis found a higher correlation (*r* = 0.973, *p* = 0.000) for 1000 mM *vs* 2000 mM than for 500 mM *vs* 1000 mM (*r* = 0.884, *p* = 0.000) and 500 mM *vs* 2000 mM (**Figures 3B,C,D**).

**Figure 4:**
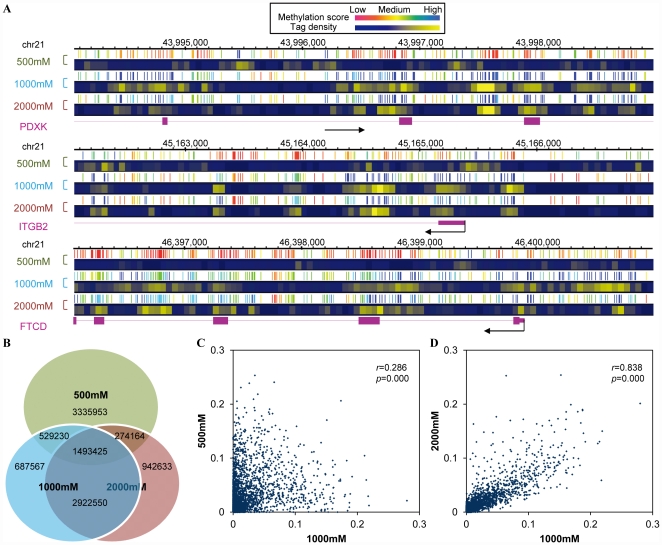
Correlation of CpG methylation score of different salt concentrations. A. Three representative regions on chromosome 21 show the methylation sites captured by 500 mM elution are distinct to those from 1000 mM and 2000 mM elution. B. A Venn diagram shows the overlap of top 20% predicted methylated CpG sites in three different elutions. 1000 mM and 2000 mM elution has an overlap of nearly 80% overlap, while only 35.9% overlap between 500 mM and 1000 mM is observed. C. and D. show the correlation of methylation probability of individual CpG site under different condition. C. shows the low correlation (*r* = 0.286, *p* = 0.000) between 500 mM and 1000 mM. D. shows the high correlation (*r* = 0.838, *p* = 0.000) between 1000 mM and 2000 mM.

The BALM analysis results of three representative regions on chromosome 21 showed that 500 mM elution captures distinct portion of methylated genomic region compared to 1000 mM and 2000 mM elution (**Figures 4A**). To quantitatively compare the detected methylated sites between three different salt concentration elutions, CpG sites with a methylation score in top 20% (5,632,772 out of 28,163,863 total CpG sites in human genome) of each salt concentration dataset were selected. A 78.4% overlap was observed between 1000 mM and 2000 mM elution, while the overlap between 500 mM and 1000 mM, 500 mM and 2000 mM are only 35.9%, 31.4% respectively (**Figures 4B**). The detected methylation score of the top 20% CpG sites in 1000 mM and 2000 mM were well correlated (*r* = 0.838, *p* = 0.000); however, there were no strong correlation between CpG sites of 500 mM and 1000 mM elution (*r* = 0.286, *p* = 0.000) (**Figure 4C, D**).

CpG islands are often important cis-regulatory elements distributed in the genome. To test the effect of CpG island methylation on gene expression genome wide, we define a CpG island methylation score as the average methylation score of all CpG dinucletides within that CpG island. We then calculated the correlation between promoter CpG island methylation score and the gene expression. Although no strong negative correlation (*r* = −0.159, *p* = 0.000) is observed, the expression difference between promoter CpG island hypermethylated group (score>0.8) of genes and hypomethylated group (score<0.2) of genes is statistically significant (student's t-test, *p* = 0.009). This result is consistent with current knowledge that gene expression is regulated at multiple levels and CpG island methylation might affect the accessibility of active or repressive transcription factors to the regulatory elements, however not directly promote or suppress gene expression [34]. Thus, we performed correlation analysis of CpG islands' DNA methylation score and DNase hyper sensitivity, which measures the accessibility of the DNA (ENCODE consortium). Genome wide analysis showed medium negative correlation (*r* = −0.454, *p* = 0.000) between these two factors (**Figure S3**).

### Optimal depth of MBD-seq

**Figure 5:**
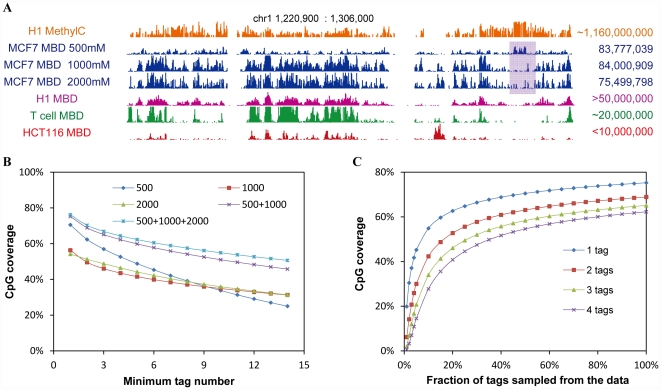
Coverage and saturation of MBD-seq experiments. A. Representative region shows the coverage of different salt concentration elution of MCF-7 MBD-seq, public available MethylC-seq data in H1 cell and MBD-seq in H1, T and HCT116 cell. Total number of tags in each dataset is listed on the right. B. Coverage analysis on five datasets, including 500 mM, 1000 mM, 2000 mM elution fraction, a double concentration (500 mM+1000 mM) and a triple concentration (500 mM+1000 mM+2000 mM) dataset, shows the double concentration dataset achieve high coverage efficiency. C. The coverage of increased fraction of random sampled tags from the double concentration datasets was plotted. The saturation curve indicates an optimal depth of MBD-seq at ∼100 million uniquely mapped tags in MCF-7 cells.

We compared the coverage the MCF-7 MBD-seq data to three public available MBD-seq data. The result shows that increased sequencing depth provides higher tags CpG coverage (**Figure 5A** **, Figure S4**). To maximize the efficiency of MBD-seq experiments, first we needed to determine an optimal combination of different salt concentration elution. Thus, we performed CpG coverage analysis [34] on five datasets, including three original datasets of MBD-seq under different salt concentration, a double concentration dataset that combined 500 mM, 1000 mM salt concentration datasets and a triple concentration dataset that combined three salt concentration datasets (**Figure 5B**). The triple concentration dataset showed increased depth because more tags were included; however, minimum improvement of coverage was observed compared to the double concentration dataset. Meanwhile, the double concentration dataset showed significant increased coverage because of the complement of 500 mM and 1000 mM datasets. After we decided the 500 mM and 1000 mM combination is the optimal elution condition, saturation analysis was performed to optimize the efficiency of sequencing depth (**Figure 5C**). Using increased fraction of random sampled data from the original 500 mM, 1000 mM combination dataset, tag coverage was calculated and plotted in **Figure 5C**. Different levels of tag coverage tend to become saturated as sequenced tag number grows. After the point 60% (approximately 100 million tags), increase in sampled tag fraction does not cause significant increase in CpG coverage. MBD-seq experiments reached optimal efficiency when using a combination of 500 mM and 1000 mM salt concentration elution and with ∼100 million unique mapped tags sequenced in MCF-7 cells. Cancer cells in general contain less genome-wide DNA methylation than their normal counterparts. This also should be taken as one of the factors when estimating optimal sequencing depth for a certain experiment.

### Resolution and efficiency of BALM

Because there are few widely accepted standalone programs available for MBD-seq data analysis, we compared the resolution of BALM with several popular peak-detection programs designed for analysis of transcription factor ChIP-seq data which is based on the same principle and shares similar procedures with MBD-seq. These programs apply similar or more advanced algorithms compared to the existing MBD-seq analysis methods most of which are based on tags density. MACS, QuEST, SISSRs and PICS were chosen because each of these programs is implemented with different algorithms and uses different statistical methods. For example, the MACS algorithm is based on shifting sequence tags towards the binding site for a certain number of base pairs then locating the binding site by calculating the summit within a peak region. QuEST identifies binding sites using a tag enrichment profile of a peak region with a Gaussian kernel. SISSRs screens binding sites in a certain window by a threshold of tags count on both forward and reverse strand calculated based on a Poisson distribution. Recently, mixture model showed advantages over several widely used programs in the ability to separate closely positioned peaks [26]. For a comprehensive comparison, we included PICS which applied a mixture t-distribution model to probabilistically infer binding sites.

**Figure 6:**
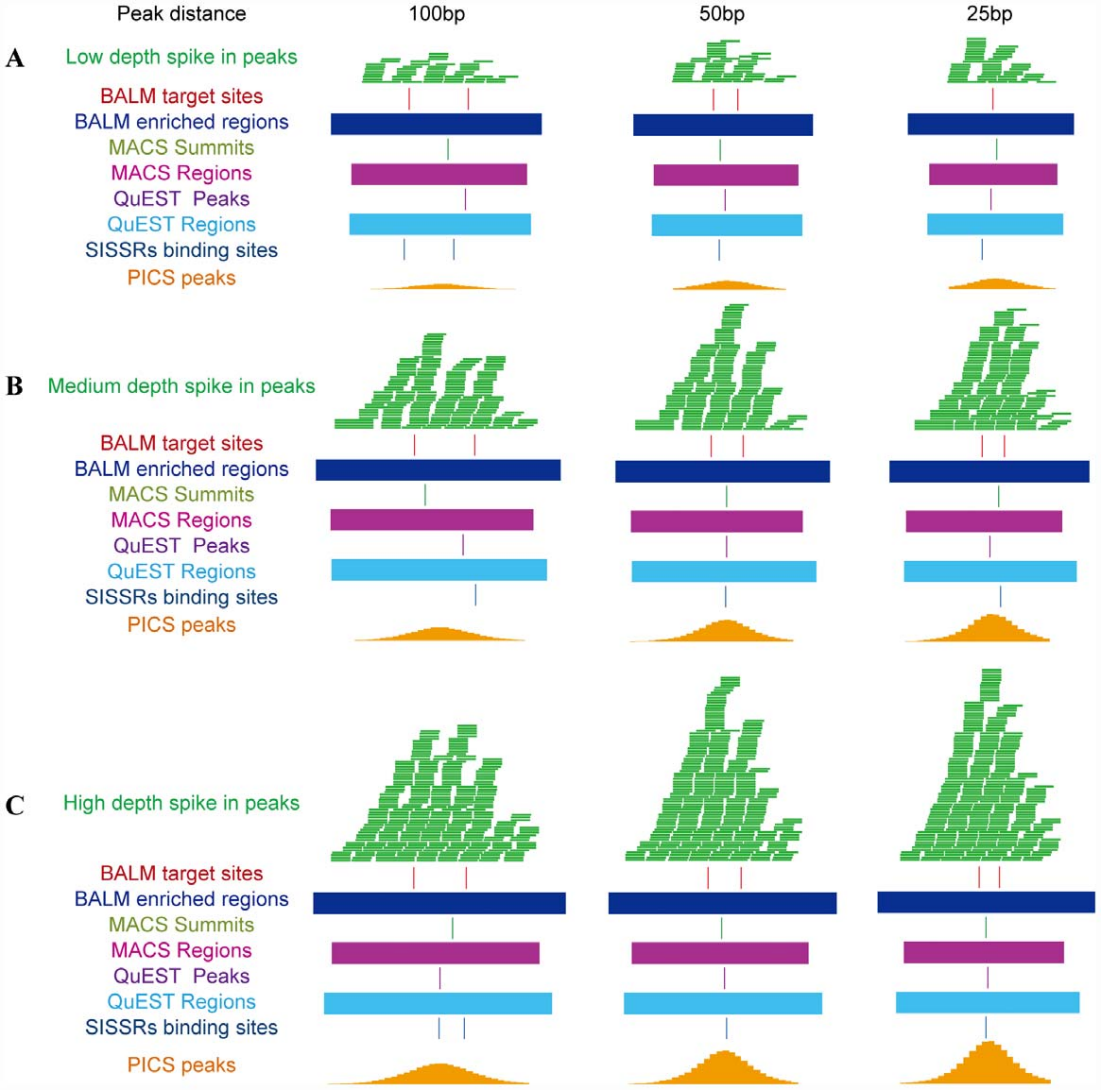
Resolution of BALM. MACS, QuEST, SISSRs, PICS and BALM's results on the spike in region with two peaks showed the ability of these programs to separate juxtaposition peaks. Three prototype peaks representing low (A), medium (B) and high (C) depth peaks respectively. In each experiment, two prototype peaks were placed 100 bp (left column), 50 bp (middle column) or 25 bp (right column) away from each another.

Firstly, to test an algorithm's ability to separate closely positioned target sites, we generated spike in data using the human transcription factor ERα dataset in MCF-7 cell [50]. Briefly, three well defined peaks, representing low depth peak, medium depth peak and high depth peak respectively, which are detectable by all five programs on chromosome 1 of the ERα dataset were inserted to random position of the genome. A second peak was then inserted at a close position to the first peak (100 bp, 50 bp, 25 bp). Five programs were then applied to detect the spike in peaks. The result shows that all of the programs identified the spike in peak region, however only BALM accurately located the two separated peaks within peak region at 50 bp resolution for low depth peaks and 25 bp resolution for medium and high depth peaks (**Figure 6**). This demonstrates the high resolution of BALM and indicates that the ability of the program to separate closely positioned peaks increases when increasing sequencing depth, which provide strong evidence supporting the high accuracy of the statistical model. The resolution limitation of BALM is at ∼50 bp for low depth regions and better resolution can be achieved at high depth regions.

Secondly, we compared the result of BALM to that of MACS and QuEST using the triple concentration MBD-seq dataset in the MCF-7 cell line. SISSRs and PICS were excluded in this comparison due to the tags number exceeding the capacity of the programs. All of the programs detected broad methylated genomic regions; however, in addition to methylated regions, BALM calculates the methylation score of each CpG dinucletides within each region. Three representative regions on Chromosome 7 show that the proposed algorithm provides users higher resolution detection and more information of DNA methylation than programs originally designed for TFBSs detection (**Figure S5**).

Applying advanced algorithm raised the concern of the program's efficiency. Furthermore, we compared the efficiency of these five algorithms by measuring computational resource consumption. BALM is relatively slow for data with small size since sophisticated statistical methods are applied; however, this algorithm is not tags number sensitive. A trend analysis (**Figure S6**) demonstrates the programs' execution time on datasets with different tag numbers. In general, the execution time of all the programs tested increases with the increase in the total number of tags. The shorter execution time for high depth datasets might be due to its C and C++ implementation as compared to MACS (Python), QuEST (Perl), SISSRs (Perl) and PICS (R).

### Clonal bisulfite sequencing validation

**Figure 7:**
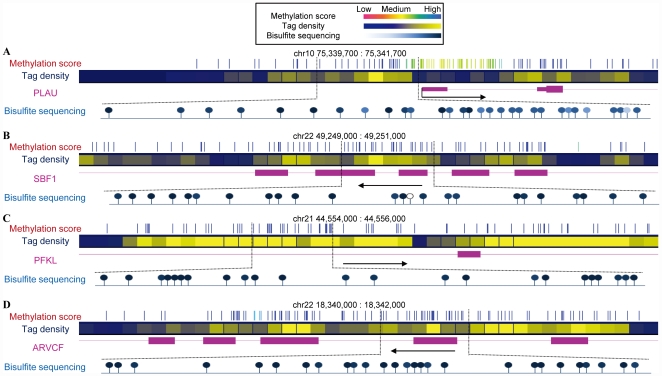
Clonal bisulfite sequencing validation. A total of 11 regions, including 2 unmethylated regions, 3 partially methylated regions and 6 heavily methylated regions with 178 CpG loci predicted from the MBD-seq in MCF-7 cell line were validated using bisulfite sequencing technique. Four representative regions at gene PLAU (A), SBF1 (B), PFKL (C) and ARVCF (D) show the accuracy of the prediction.

To validate methylation sites identified from MBD-seq and assess the resolution and accuracy of BALM, we performed standard clonal bisulfite sequencing in randomly selected regions in MCF-7 cells. 11 regions, including 2 unmethylated, 3 partially methylated and 6 fully methylated regions with both dense and spotty CpG sites. Comparing a total of 178 CpG di-nucleotide loci's bisulfite sequencing results to the corresponding methylation score produced by BALM yielded a Pearson correlation coefficient *r* = 0.879 (*p* = 0.000) The results demonstrated the prediction of DNA methylation by BALM is accurate and reliable not only in sparse but also in dense CpG regions (**Figure 7** **,** **Figure S7, S8, S9**).

## Discussion

MBD-seq is widely used as a cost efficient method to investigate genome wide methylation pattern. In this study, we attempted to determine the optimal condition for the MBD-seq experiment. We performed high depth MBD-seq under three different elution salt concentrations (500 mM, 1000 mM, 2000 mM) in MCF-7 cell line. The analysis indicates that different salt concentrations can be used in MBD-seq to yield distinctive populations of methylated DNA fragments. The result shows that with ∼100 million unique mapped tags (approximately five lanes for a GAII sequencer) from 500 mM and 1000 mM elutes the coverage of the MBD-seq data become close to a saturation point. MBD protein's affinity to methylated DNA is enhanced as the density of methylated CpG sites increases [34]. This makes MBD-seq a highly effective method to measure the methylation status of CpG islands, within which CpG sites are densely distributed. As many of the studies are focused on CpG island methylation, accurate measurement can be achieved at a lower sequencing depth.

Interestingly, a medium negative correlation of CpG island methylation score and DNase hyper sensitivity is observed. However, this result does not identify a causal relationship between DNA methylation and chromatin status. H3K4 methylation has been reported as an active histone modification mark on the chromatin affecting the accessibility of the *cis*-element. Relationship between H3K4 methylation and DNA methylation may be interesting for further study given the negative correlation between DNA methylation and chromatin accessibility.

To finely determine the methylation level of each CpG dinucleotides in the genome, we developed a statistic model named BALM. There are several noteworthy features which increase the accuracy of the presented algorithm.

Firstly, unlike TFBSs, methylated CpG dinucleotides are highly abundant in the genome and densely distributed. The new program is capable of distinguishing two closely positioned target sites by applying EM algorithm to approximate a BALM mixture. Indeed, the proposed algorithm increases the resolution of MBD-seq from 150 bp to 50 bp and up to 25 bp in signal highly enriched regions [34]. A methylation score is calculated for each genomic CpG site based on the statistic model. This score is an effective indication of the probability of the position being methylated or not. Therefore, it allows users to interpret the data in a more appropriate and effective way.

Secondly, as many efforts are made to understand the abnormal transcriptional regulation and aberrant epigenetic event in cancer development, more high-throughput data derived from cancer model cell lines are available. The majority of these model cell lines' genomes are disrupted, for example, MCF-7, LNCaP, K562 etc. Another important feature of the program is by using a genome region amplification index (*GRAI*), it increases the threshold of the weighted enrichment as well as the number of background noise tags simulated to better control the *FDR* in amplified genomic regions. For example, several genomic regions on the q arm of chromosome 20 in MCF-7 cell line were reported to have a high amplification number [51]. To access the bias control provided by the *GRAI*, the index of 20q generated based on the input data of MBD-seq was plotted with the result from the study of Volik S et al [51] (**Figure S10**). The comparison showed that the *GRAI* precisely reflected the copy number changes of different genomic regions.

In summary, we produced extra high sequencing depth MBD-seq data in MCF-7 cell line and determined the optimal parameters for MBD-seq experiment. The new algorithm was specifically designed to address the emerging demand of higher resolution detection of densely distributed CpG dinucleotides' methylation level. Through validation using clonal bisulfite sequencing, this study demonstrates that the combination of BALM and MBD-seq could serve as a powerful method to capture genome wide DNA methylation profile with high efficiency, high resolution and low cost.

## Materials and Methods

### Methylated DNA enrichment and high-throughput sequencing (MBD-seq)

Purified genomic DNA from the breast cancer cell-line MCF-7 (BioChain, Hayward, CA) was fragmented using an S2 non-contact Adaptive Focused Acoustics™ ultrasonicator (Covaris, Woburn, MA) as described in the SOLiD™ 3 fragment library protocol to generate randomly fragmented DNA of 50–350 bp in length. Fragmented DNA was then subjected to MethylMiner™ methylated DNA kit enrichment (which uses a recombinant form of the human MBD2 protein) according to the manufacturer's protocol and two methylated fractions (500 mM and 1000 mM salt eluates) were isolated. The recovered eluted mass of DNA was 6.9% (3.47 mg) of the total mass loaded (50 mg). Subsequent elution at very high NaCl concentration (3.5 M) followed by digestion with proteinase K shows that less than 10% of the captured DNA remains on the beads after elution with 1 M NaCl. Separately, 25 µg of fragmented genomic DNA was enriched with a MethylMiner™ kit and eluted as a single fraction with buffer containing 2000 mM NaCl. Unenriched genomic DNA fragments, 500 mM, 1000 mM, and 2000 mM DNA fractions were then used to construct standard fragment libraries using a combination of adaptor ligation and nick translation (SOLiD™ Fragment Library Construction Kit, Invitrogen). Library DNA, was size-selected (inserts were ∼100–200 bp) by gel-purification from 2% agarose E-Gel® EX gels prior to PCR amplification, attachment to beads, and emulsion PCR. Libraries were sequenced in 4-well deposition chambers on a SOLiD™ 3 Analyzer and sequenced tags corresponding to 50 base lengths were obtained. The resulting tag sequence csfasta and quality files were aligned to the human genome (NCBI Build 36.1, UCSC Hg18) using the Bowtie mapping software [35].

### Clonal Sanger bisulfite sequencing

2.5 µg of MCF-7 cell line genomic DNA (Biochain) was bisulfite converted with MethylCode Bisulfite Conversion Kit (Invitrogen) in 5 reactions at 500 ng scale each. PCR amplification of 50 ng equivalent of starting amount of converted DNA was performed with C to T conversion specific primers (avoiding CpG regions) with the following PCR mix: 2 units of AccuPrime Taq DNA Polymerase High Fidelity enzyme (Invitrogen), 1× AccuPrime PCR Buffer II, 0.2 µM each primer final, in 100 µl final volume, with cycling conditions as follows: initial denaturation at 94°C 2 min., 40 cycles (denature at 94°C 15 sec., anneal at 53–62°C 30 sec., extend at 68°C 1 min.), final extension at 68°C 5 min., 4°C hold. Detailed information about primers used for each amplicon is listed in **Table S3**. PCR Products (1–2 µl) were cloned into pCR4-TOPO sequencing vector (Invitrogen), and transformed into TOP10 chemically competent *E.Coli.* (Invitrogen). Transformation was then plated on LB+100 Ampicillin plates and incubated at 37°C overnight. Up to 20 individual colonies of each amplicon were grown in 1 ml BRM+100 µg/ml cultures overnight in a 96 well culture block at 37°C at 300 RPM. Plasmid DNA was isolated using PureLink HQ 96 Plasmid Purification Kit (Invitrogen), and 1 µg of each clone was sequenced using M13 Reverse primer from TOPO kit (Invitrogen) by Sanger sequencing technology.

### Public datasets

MethylC-seq data in H1 human embryonic stem cells [21], MBD-seq data in H1 human embryonic stem cells [34], human T cell[33] and HCT116 human colon cancer cells [18]. ChIP-seq data for human transcription factors CTCF in CD4+ T cell [5], FOXA1 in MCF-7 cell [23], ERα in MCF-7 cell [50], and NRSF in Jurkat lymphoblast cell [6] were downloaded for evaluating the performance of the program. All datasets are available at http://motif.bmi.ohio-state.edu/BALM/.

### BALM algorithm

#### Initial scan for target sites

In the first step, an average fragment length is determined by taking the average distance between the mean position of the tags on the forward strand and reverse strand of top enriched bimodal pileup regions [37]. Then, all tags are shifted towards the mid-point by half of the average fragment length. A sliding window of 50 bp is used to scan regions that have a higher fragments count than a predetermined threshold weighted by the *GRAI* (Text S1). Within each of these regions, a target site is calculated by taking the average of the positions of the fragments in this region. Shifted tag positions are used for the initial detection of target sites, while the original tag positions are used in constructing the model and subsequent analysis.

#### Parameter estimation of the BALM model

The density function for an asymmetric Laplace distribution (ALD) is,
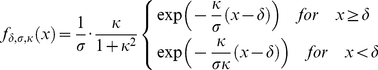
(1)Two steps are followed to obtain the maximum likelihood estimators of the ALD [38], [39],

1. Given sample size *n*, find *x_m_*, *1≤m≤n* that minimize the function 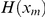 ,

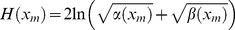
(2)where 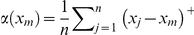, 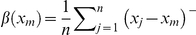,
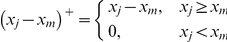


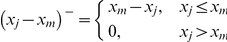
In above equations, *x_j_* is the *j*th element of *x* and *x_m_* is the element that minimize function *H*(*x_m_*).

1. set

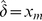
(3)

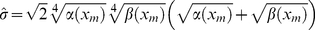
(4)

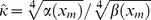
(5)


#### Maximization of the likelihood of tags within enriched regions using the EM algorithm

Within a signal enriched region, multiple target sites might exist. These sites are estimated by maximizing the likelihood of the given tags using a BALM mixture,
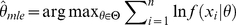
(6)Where *f* is the probability density function of the BALM mixture and *n* is the number of tags.

The detailed procedure of EM algorithm is discussed in Text S1 in the EM algorithm section.

#### Determination of the best mixture model

Bayesian Information Criterion (*BIC*) is used to determine the number of components (target sites) within a signal enriched region.
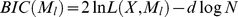
(7)where 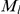 is the model being tested, 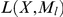 is the log likelihood that the given sample is generated from model 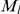, 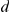 is the number of free parameters in model 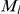 and 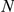 sample size, in this case, tags number in a given enriched region.

#### Calculation of weighted enrichment level

The weight of a tag with respect to a target site is determined by the relative position of that tag to the target site. The weight is proportional to a probability P modeled by the BALM described above. The weighted enrichment score of each position is calculated as follows,
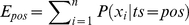
(8)where *ts* represents target site, *pos* represents a genomic position and *n* is the total number of observations around that target site.

#### Generation of the genome region amplification index (GRAI)

The *GRAI* is an indicator of the copy number of a certain genomic region. When input data are available, a local background enrichment level can be calculated by counting the tags that mapped to that local region. The index is constructed by taking the ratio of the local background enrichment to the genome background enrichment.
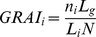
(9)where *n_i_ denotes* the input tags number in the *i*th region, *L_g_* denotes the genome size, *L_i_* represents the length of the *i*th region and *N* is the total number of input tags.

### Estimation of False Discovery Rate (FDR)

Monte-Carlo simulation is performed to generate simulated data and compute *FDR*. Each dataset includes simulated peaks and background noise tags based on the real MBD-seq data [37].

### Program implementation

BALM is implemented in C and C++. The source code is platform independent and was compiled and tested in Linux Fedora10, OS X with the gcc compiler and Windows XP, Vista with Microsoft Visual C++ 9.0 compiler. The documentation, source code and compiled binaries for Linux, OS X, Windows XP and Vista can be downloaded at http://motif.bmi.ohio-state.edu/BALM/download.

The program takes inputs of various tag information file formats, such as BED, ELAND, EXTENDED ELAND, GFF, SAM and Bowtie alignment file. The default input file format is ELAND. It also provides an option for non standard file formats, which allows users to specify the columns containing chromosome name, start, end, and strand of a tag (e.g. –n 0 1 2 5). If control data are available, the -c option specifies the control file name (e.g. -c XXX_IgG.txt). –c option can also be used to compare different samples.

Some of the most commonly used genome assemblies are included, e.g. Human (hg18, hg19) and Mouse (mm8, mm9). The default assembly is hg18. For genomes that are not included, users need to provide genome information, such as size, chromosome number, and length of chromosomes. By default, pericentromeric and repetitive region are excluded from the output using a species specific repetitive region table; however, this filter off can be turned off if desired.

In addition, options –o and -W allow the program to generate files with fragment enrichment levels across the genome, in variable step and fixed step WIG format respectively after the tags have been shifted. These files can be easily visualized by any genome browser taking the WIG format file as input, such as the UCSC genome browser [52] and the Integrated Genome Browser [53]. Tools that can convert files between different file formats are integrated in the program.

A user friendly GUI for BALM is provided to help biologist specifying parameter for running the program (Text S1).

## Supporting Information

Figure S1 **Fragment length distribution after sonication followed by size selection from an unpublished paired-end sequencing data.**
(PDF)

Figure S2 **Tags distribution around transcription factor binding sites.**
(PDF)

Figure S3 **Correlation between CpG island methylation score and DNase hyper sensitivity in MCF-7 cell line.**
(PDF)

Figure S4 **Coverage and saturation of MBD-seq experiments.**
(PDF)

Figure S5 **A comparison of BALM with MACS, QuEST on the result of MBD-seq data in MCF-7 cell.**
(PDF)

Figure S6 **Comparison of algorithm efficiency in terms of execution time.**
(PDF)

Figure S7 **Validation of MBD-seq using bisulfite sequencing technique, ESPN, PLEKHG5, HOXA11, PLAU.**
(PDF)

Figure S8 **Validation of MBD-seq using bisulfite sequencing technique, MC5R, PIK3C3, KIAA0427, C18ORF24.**
(PDF)

Figure S9 **Validation of MBD-seq using bisulfite sequencing technique, PFKL, ARVCF, SBF1.**
(PDF)

Figure S10 **Comparison between GRAI and the result of end-sequencing profiling technique developed by Volik et al.**
(PDF)

Table S1 **Summary of MDB-Seq tags from different elution.**
(DOC)

Table S2 **Estimated BALM parameters.**
(DOC)

Table S3 **PCR primer.**
(DOC)

Text S1 **Supplementary methods.**
(PDF)
