# Supplementary material for: High Resolution Detection and Analysis of CpG Dinucleotides Methylation Using MBD-Seq Technology
Source: PLoS One. 2011 Jul 11;6(7):e22226. doi: 10.1371/journal.pone.0022226 (PMC3136941; doi:10.1371/journal.pone.0022226)

Figure S1. Fragment length distribution after sonication followed by size selection from an unpublished paired-end sequencing data produced in our lab.

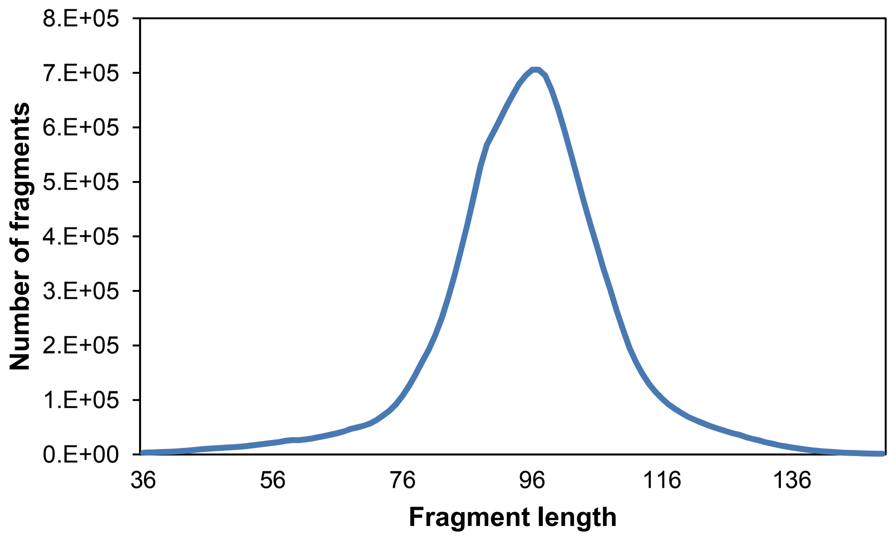

Supplement: Figure S1 — Fragment length distribution after sonication followed by size selection from an unpublished paired-end sequencing data. (PDF) [file pone.0022226.s001.pdf]
