# Supplementary figures and images for: High Resolution Detection and Analysis of CpG Dinucleotides Methylation Using MBD-Seq Technology
Source: PLoS One. 2011 Jul 11;6(7):e22226. doi: 10.1371/journal.pone.0022226 (PMC3136941; doi:10.1371/journal.pone.0022226)

**Figure S2.** Tags distribution around transcription factor binding sites

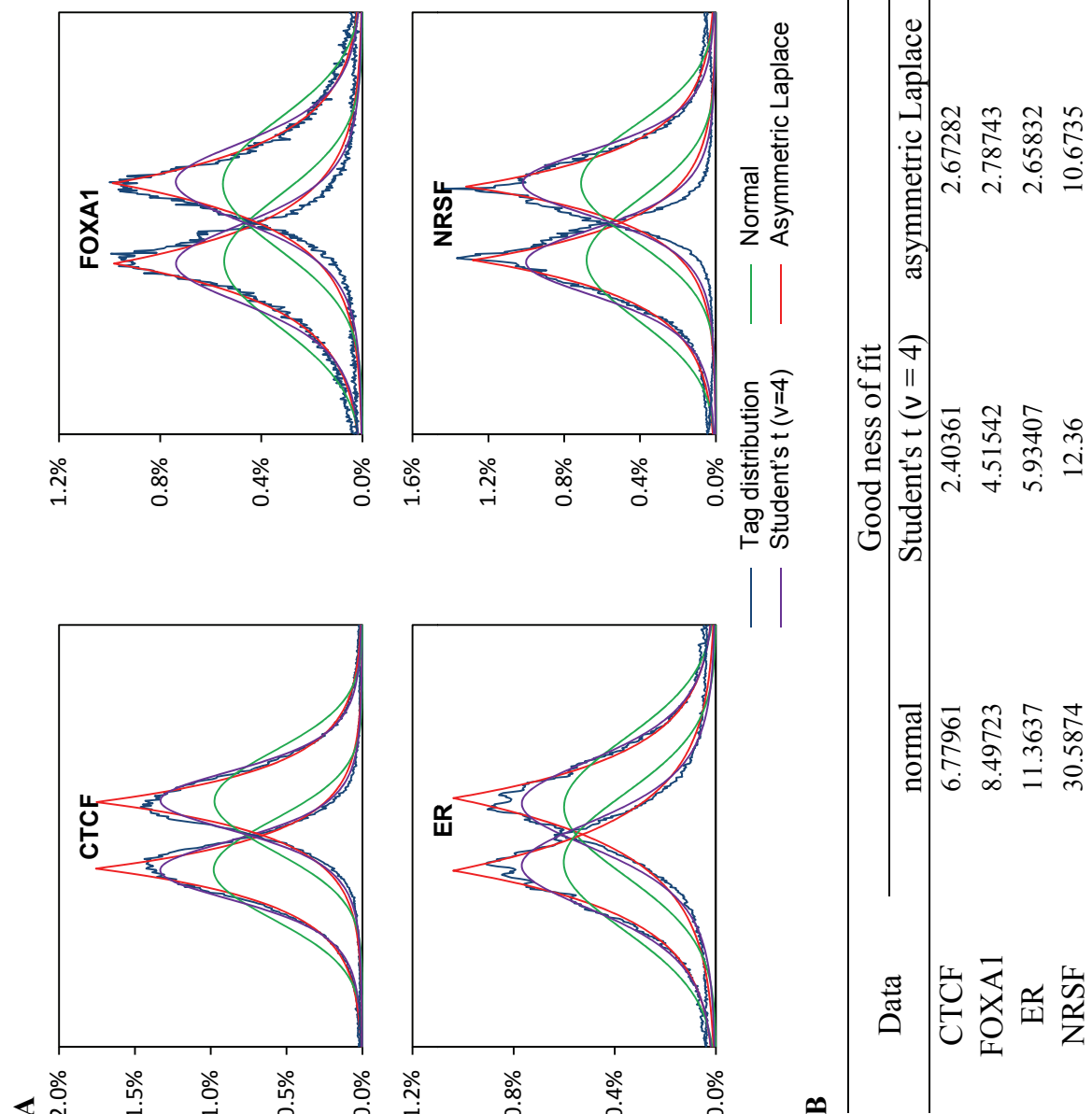

Supplement: Figure S2 — Tags distribution around transcription factor binding sites. (PDF) [file pone.0022226.s002.pdf]

**Figure S4.** Coverage and saturation of MBD-seq experiments.

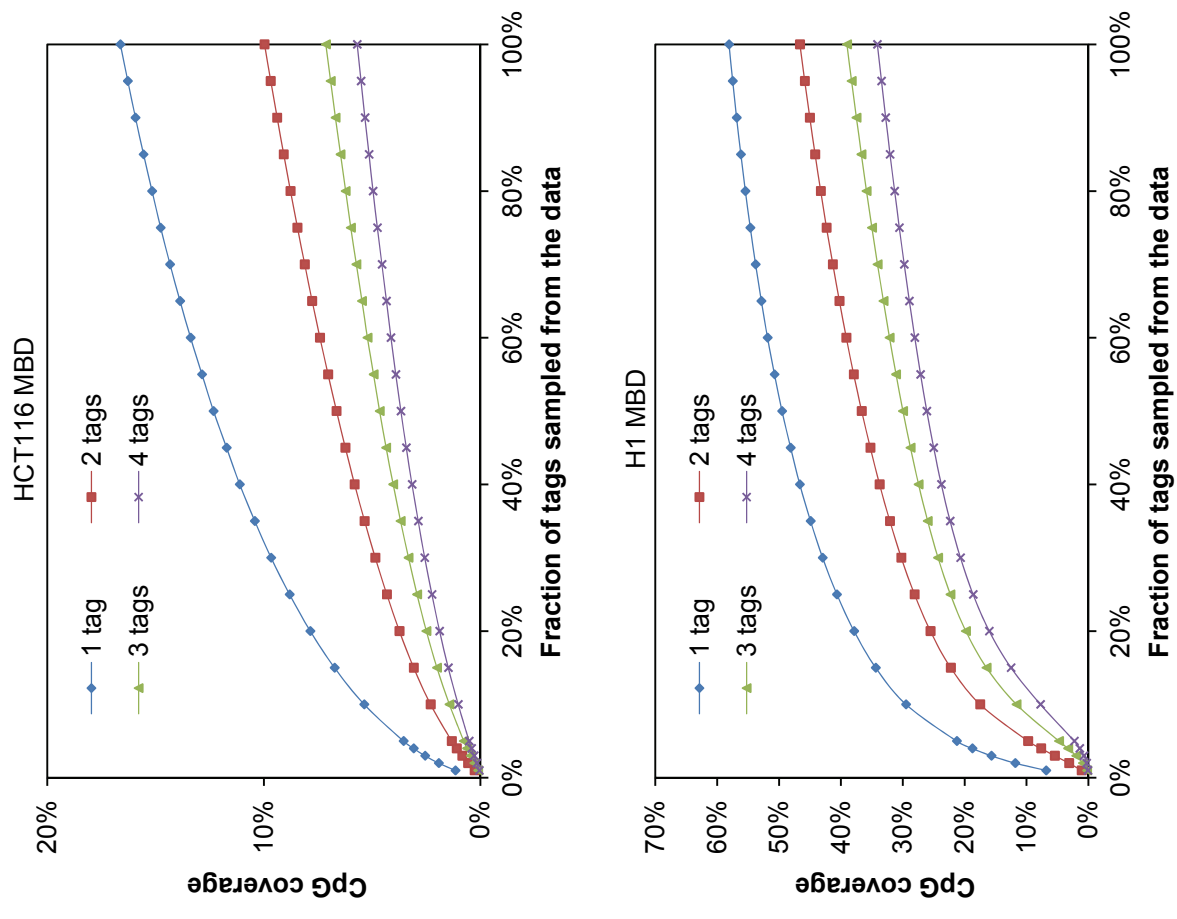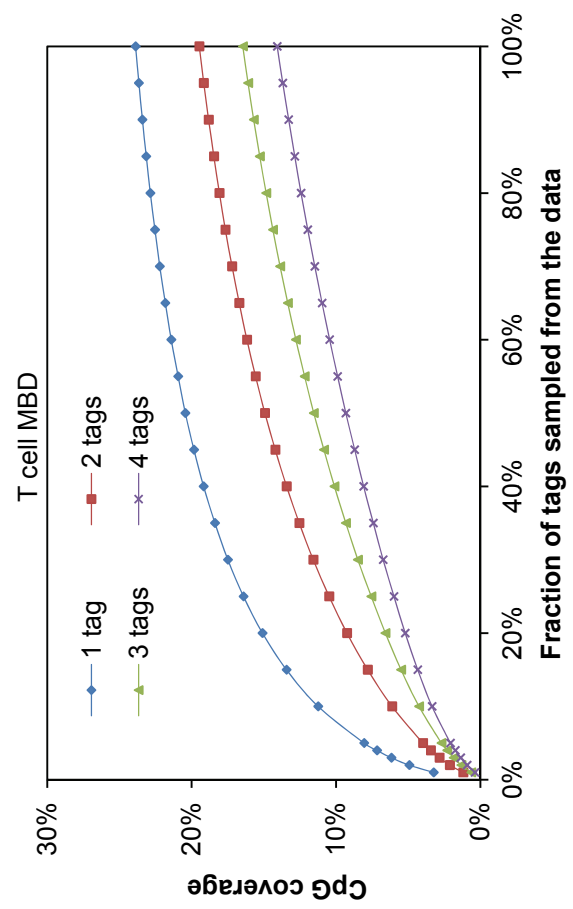

Supplement: Figure S4 — Coverage and saturation of MBD-seq experiments. (PDF) [file pone.0022226.s004.pdf]

**Figure S6.** Comparison of algorithm efficiency in terms of execution time.

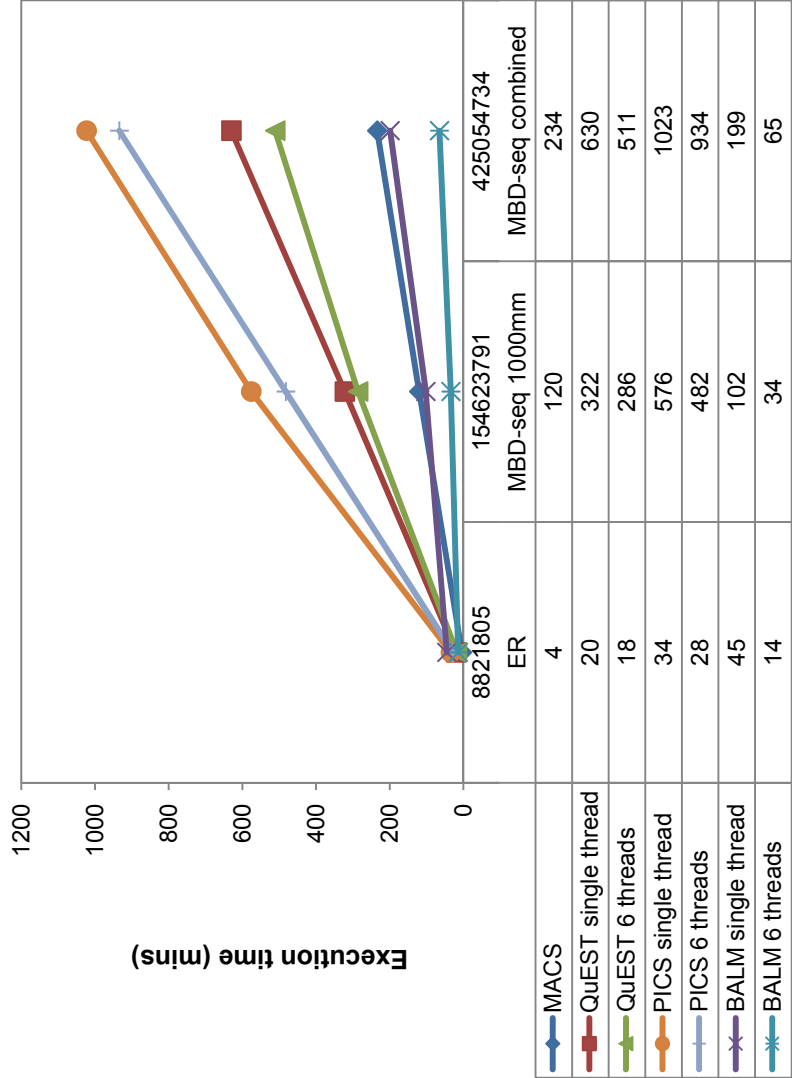

Supplement: Figure S6 — Comparison of algorithm efficiency in terms of execution time. (PDF) [file pone.0022226.s006.pdf]

**Figure S8.** Validation of MBD-seq using bisulfite sequencing technique, PLAU.

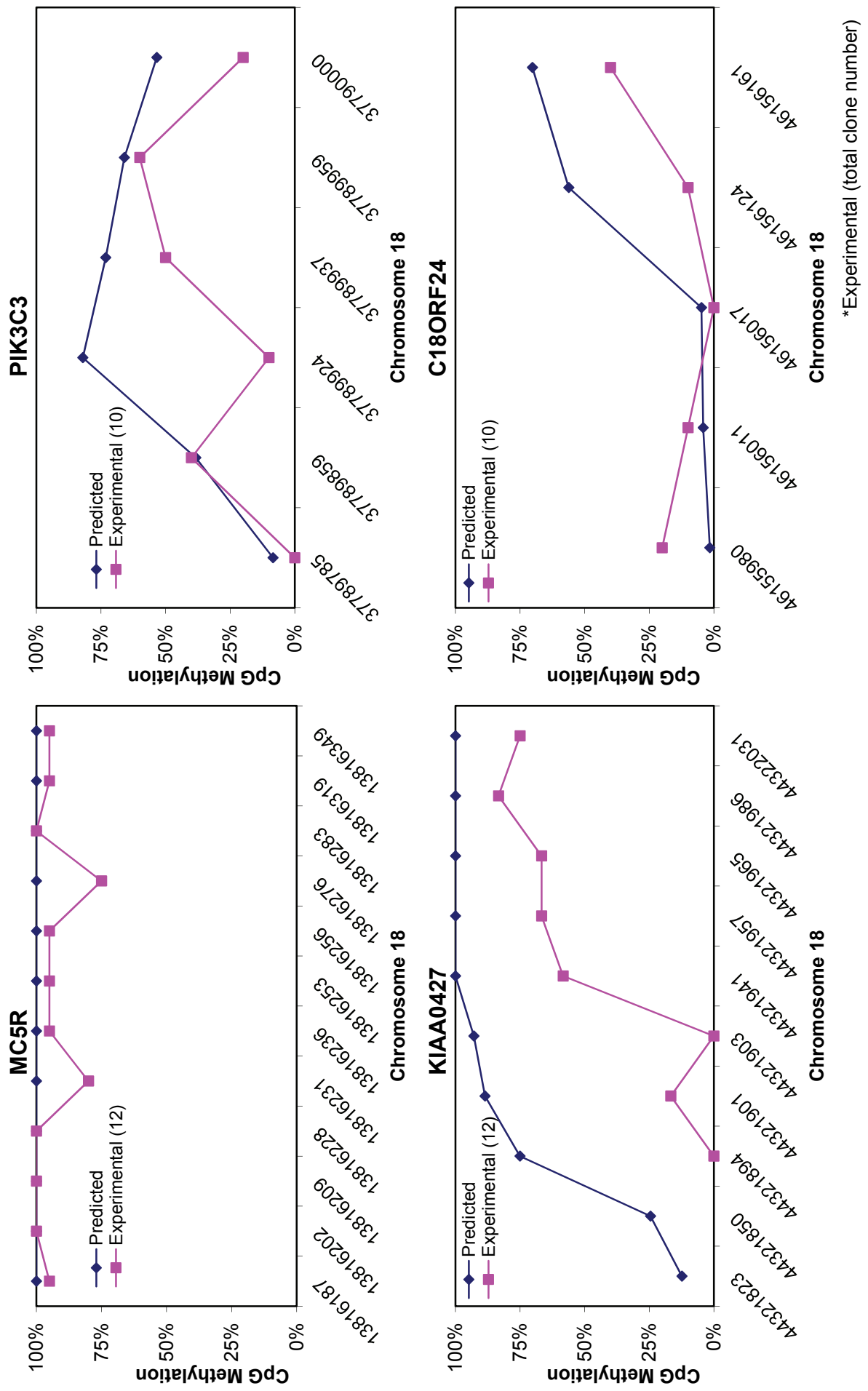

Supplement: Figure S8 — Validation of MBD-seq using bisulfite sequencing technique, MC5R, PIK3C3, KIAA0427, C18ORF24. (PDF) [file pone.0022226.s008.pdf]
