# Supplementary material for: High Resolution Detection and Analysis of CpG Dinucleotides Methylation Using MBD-Seq Technology
Source: PLoS One. 2011 Jul 11;6(7):e22226. doi: 10.1371/journal.pone.0022226 (PMC3136941; doi:10.1371/journal.pone.0022226)

Figure S3. Correlation between CpG island methylation score and DNase hyper sensitivity in MCF-7 cell line.

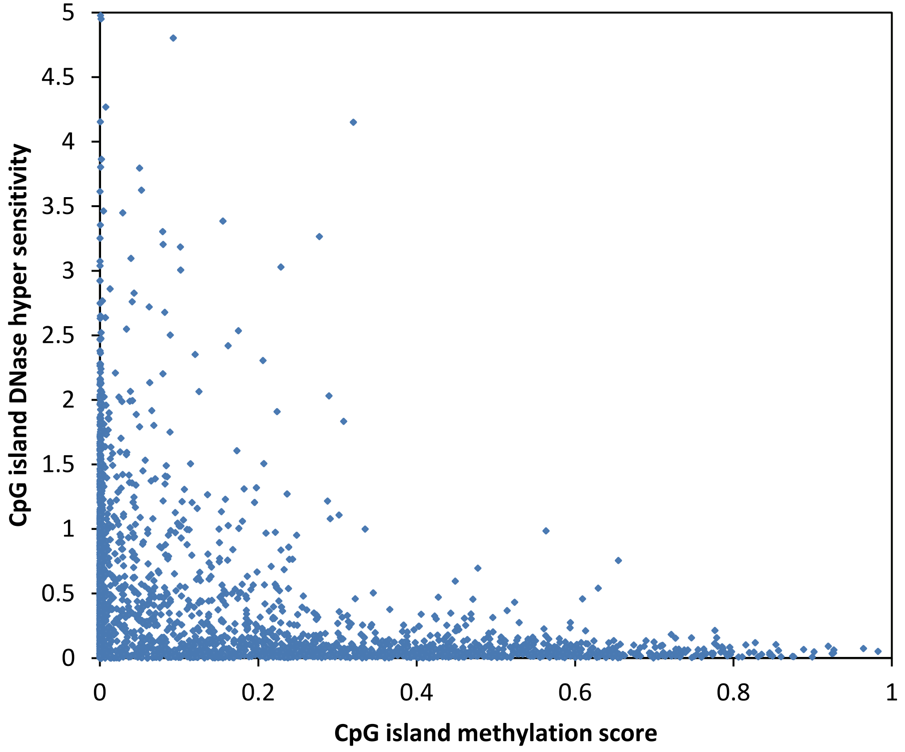

Supplement: Figure S3 — Correlation between CpG island methylation score and DNase hyper sensitivity in MCF-7 cell line. (PDF) [file pone.0022226.s003.pdf]
