# Supplementary material for: High Resolution Detection and Analysis of CpG Dinucleotides Methylation Using MBD-Seq Technology
Source: PLoS One. 2011 Jul 11;6(7):e22226. doi: 10.1371/journal.pone.0022226 (PMC3136941; doi:10.1371/journal.pone.0022226)

Figure S5. A comparison of BALM with MACS, QuEST on the result of MBD-seq data in MCF-7 cell. All three programs similarly detected broad methylation regions. QuEST distinguished different summits in the large region. With facility provided, MACS is also likely to detect sub-peaks in this region; however, BALM further finely estimate the degree of each CpG dinucletide being methylated by the EM and BIC algorithm.

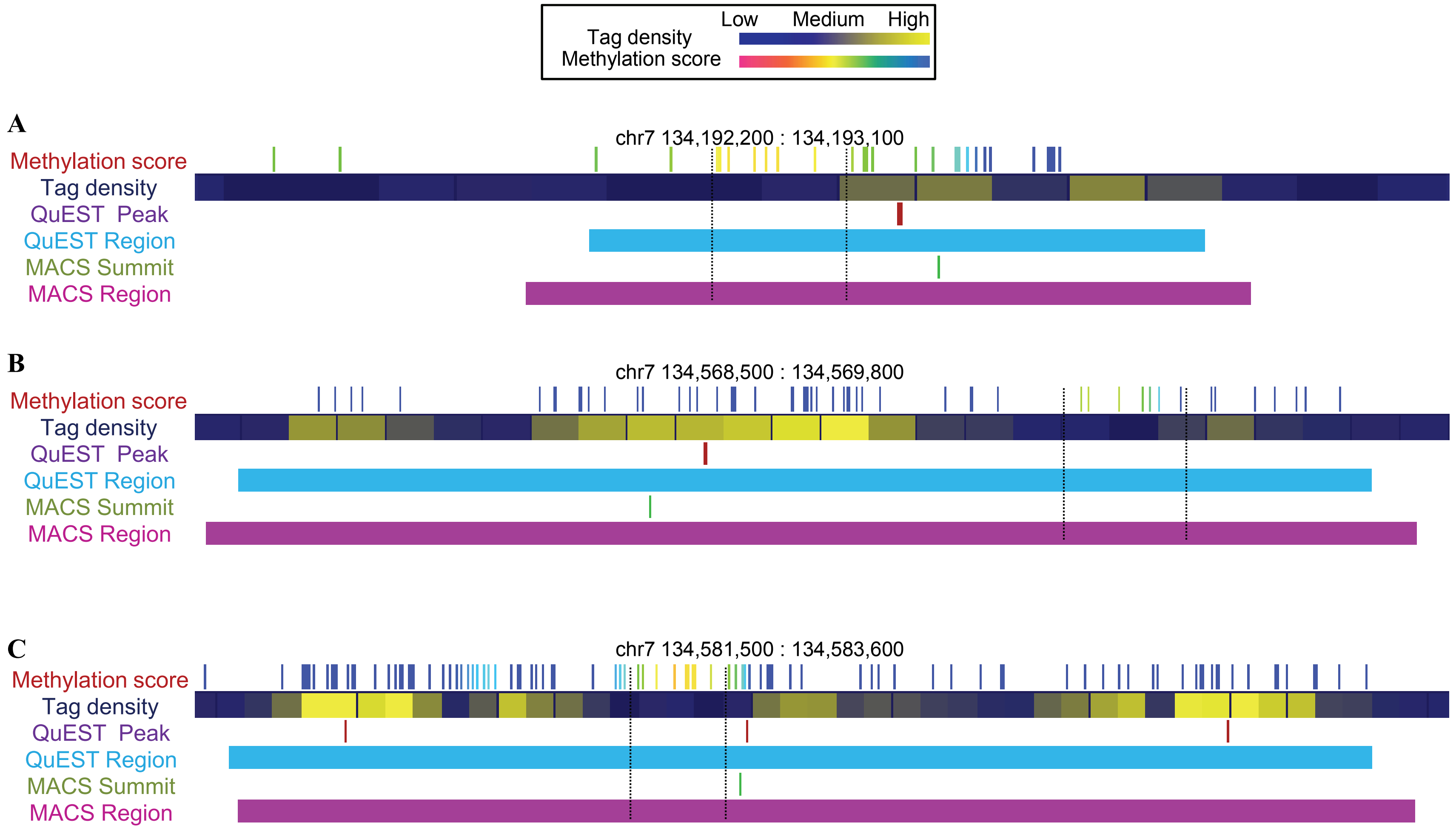

Supplement: Figure S5 — A comparison of BALM with MACS, QuEST on the result of MBD-seq data in MCF-7 cell. (PDF) [file pone.0022226.s005.pdf]
