# Supplementary material for: High Resolution Detection and Analysis of CpG Dinucleotides Methylation Using MBD-Seq Technology
Source: PLoS One. 2011 Jul 11;6(7):e22226. doi: 10.1371/journal.pone.0022226 (PMC3136941; doi:10.1371/journal.pone.0022226)
Supplement: Figure S7 — Validation of MBD-seq using bisulfite sequencing technique, ESPN, PLEKHG5, HOXA11, PLAU. (PDF) [file pone.0022226.s007.pdf]

Figure S7. Validation of MBD-seq using bisulfite sequencing technique.

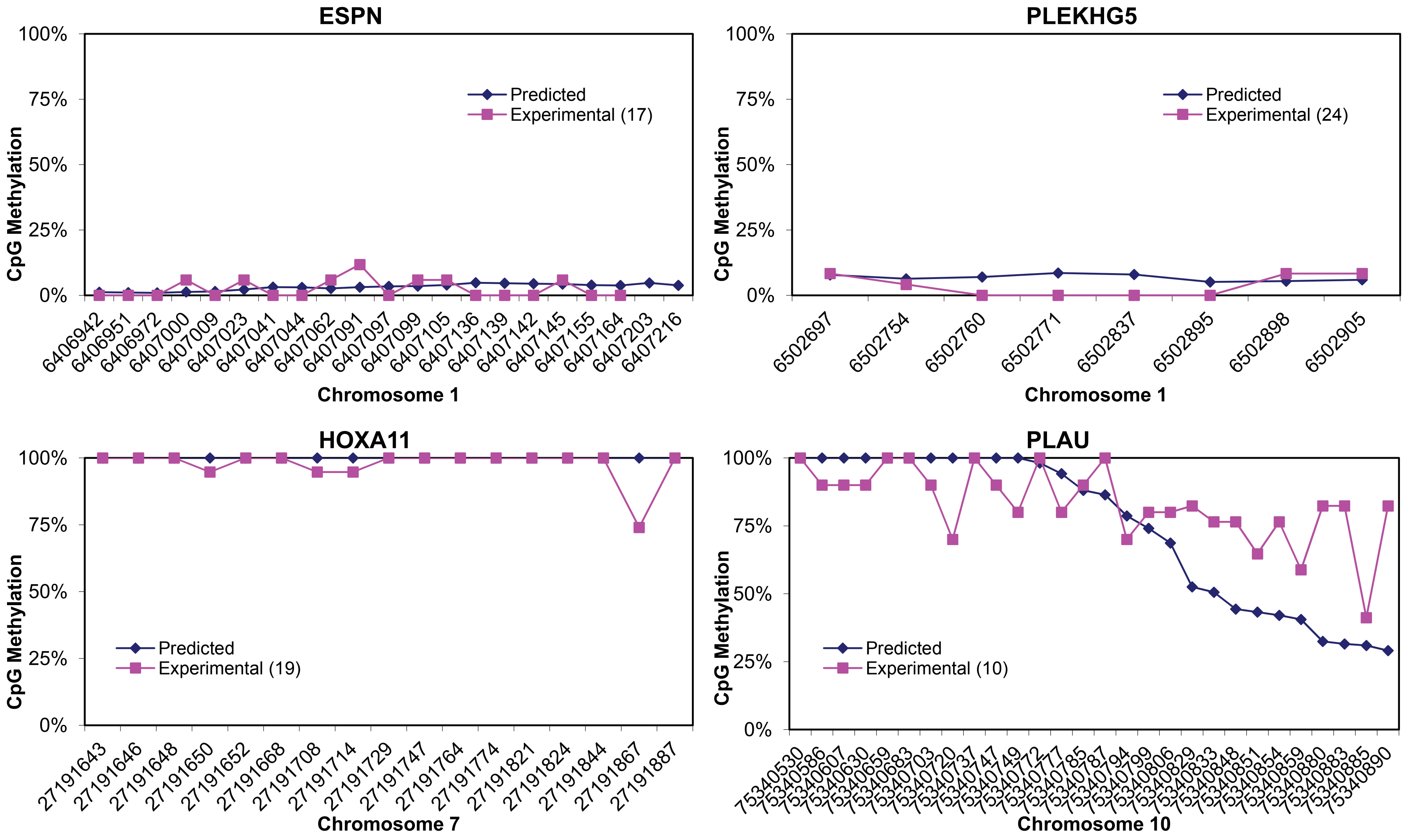

*Experimental (total clone number)
