# Supplementary material for: High Resolution Detection and Analysis of CpG Dinucleotides Methylation Using MBD-Seq Technology
Source: PLoS One. 2011 Jul 11;6(7):e22226. doi: 10.1371/journal.pone.0022226 (PMC3136941; doi:10.1371/journal.pone.0022226)
Supplement: Figure S9 — Validation of MBD-seq using bisulfite sequencing technique, PFKL, ARVCF, SBF1. (PDF) [file pone.0022226.s009.pdf]

**Figure S9.** Validation of MBD-seq using bisulfite sequencing technique, PLAU.

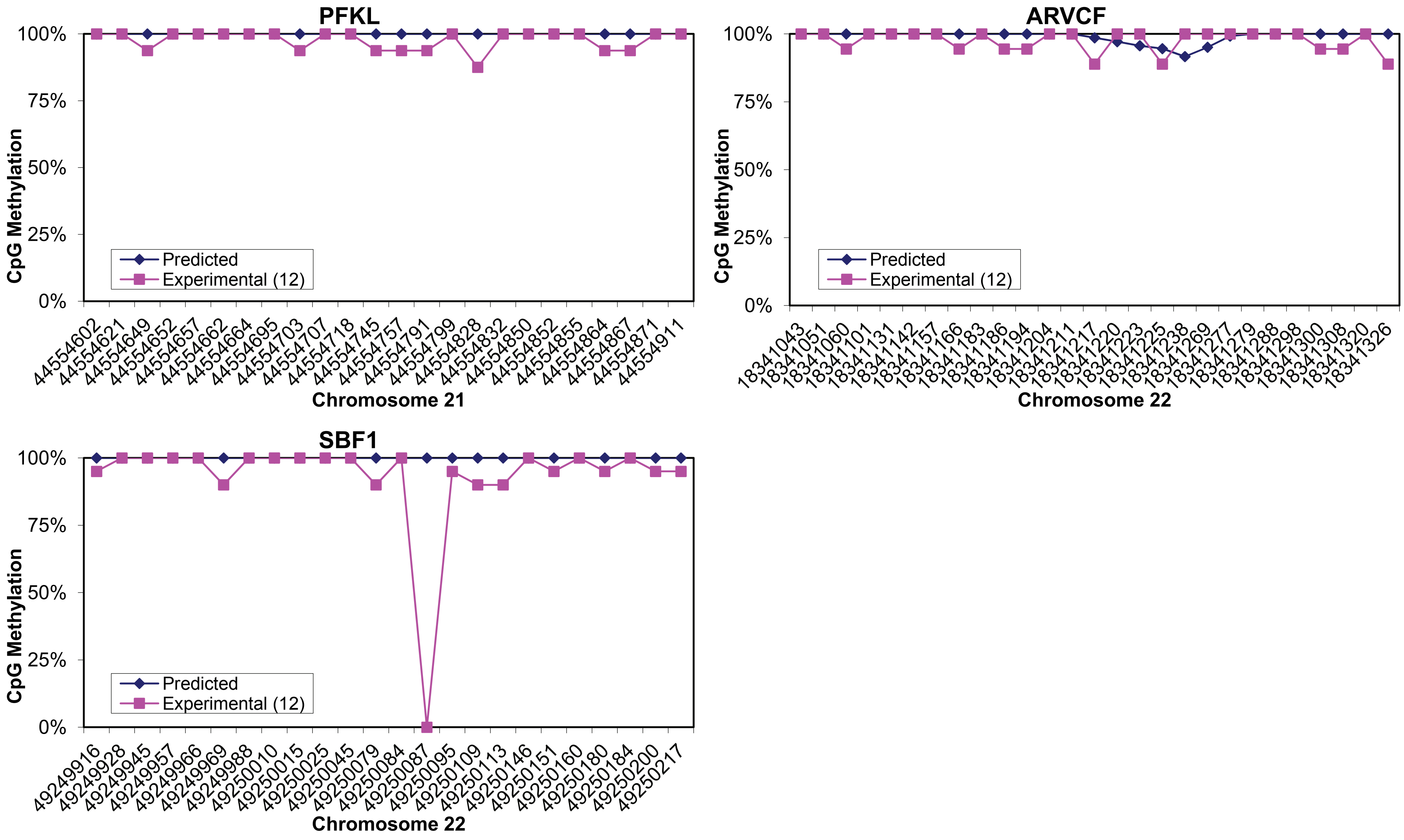


*Experimental (total clone number)
