# Supplementary material for: High Resolution Detection and Analysis of CpG Dinucleotides Methylation Using MBD-Seq Technology
Source: PLoS One. 2011 Jul 11;6(7):e22226. doi: 10.1371/journal.pone.0022226 (PMC3136941; doi:10.1371/journal.pone.0022226)

Figure S10. Comparison between GRAI of MCF-7 cell line based on the MBD-Seq input data and the result of end-sequencing profiling technique developed by Volik et al..

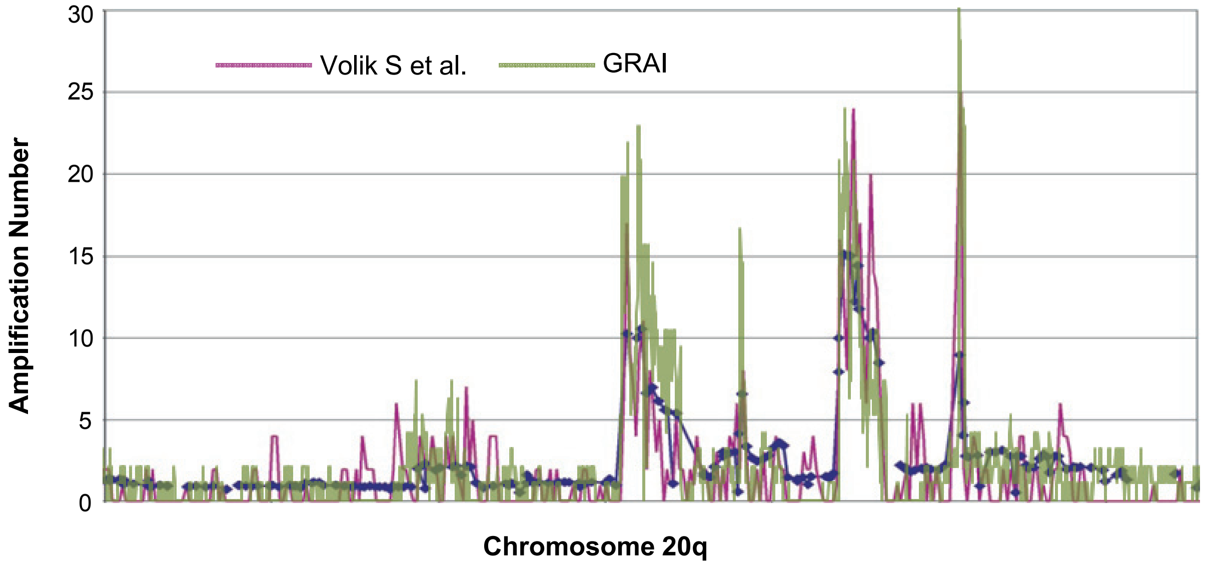

Supplement: Figure S10 — Comparison between GRAI and the result of end-sequencing profiling technique developed by Volik et al. (PDF) [file pone.0022226.s010.pdf]
