# Supplementary material for: High Resolution Detection and Analysis of CpG Dinucleotides Methylation Using MBD-Seq Technology
Source: PLoS One. 2011 Jul 11;6(7):e22226. doi: 10.1371/journal.pone.0022226 (PMC3136941; doi:10.1371/journal.pone.0022226)
Supplement: Table S1 — Summary of MDB-Seq tags from different elution. (DOC) [file pone.0022226.s011.doc]

**Table S1.** Summary of MDB-Seq tags from different elution

| Salt Concentration | Total Mapped Tags | Unique Mapped Tags |
| --- | --- | --- |
| 500mm | 130,965,175 | 83,777,039 |
| 1000mm | 154,623,791 | 84,000,909 |
| 2000mm | 139,465,768 | 75,499,798 |
| Input | 72,970,099 | 50,840,361 |
