# Supplementary material for: High Resolution Detection and Analysis of CpG Dinucleotides Methylation Using MBD-Seq Technology
Source: PLoS One. 2011 Jul 11;6(7):e22226. doi: 10.1371/journal.pone.0022226 (PMC3136941; doi:10.1371/journal.pone.0022226)
Supplement: Table S2 — Estimated BALM parameters. (DOC) [file pone.0022226.s012.doc]

**Table S2.** Estimated BALM parameters

| Data | Strand | Parameters | | |
| --- | --- | --- | --- | --- |
|  |  | θ | σ | κ |
| MBD in MCF7 | forward | -45 | 78.6999 | 0.854124 |
|  | reverse | 45 | 77.5207 | 1.12074 |
|  |  |  |  |  |
| MBD in H1 | forward | -77 | 83.9296 | 1.22654 |
|  | reverse | 77 | 88.5777 | 0.779785 |
|  |  |  |  |  |
| MBD in T cell | forward | -53 | 73.0629 | 0.92573 |
|  | reverse | 54 | 72.0422 | 1.0732 |
|  |  |  |  |  |
| MBD in HCT116 | forward | -47 | 70.1349 | 0.897699 |
|  | reverse | 47 | 69.6271 | 1.0302 |
|  |  |  |  |  |
| CTCF | forward | -27 | 40.2385 | 1.01702 |
|  | reverse | 28 | 40.3395 | 0.986449 |
|  |  |  |  |  |
| FOXA1 | forward | -45 | 71.8979 | 0.975041 |
|  | reverse | 45 | 71.591 | 1.00957 |
|  |  |  |  |  |
| ER | forward | -35 | 67.7709 | 0.917369 |
|  | reverse | 36 | 67.7936 | 1.09561 |
|  |  |  |  |  |
| NRSF | forward | -36 | 55.1012 | 1.00033 |
|  | reverse | 36 | 53.6163 | 0.959186 |
