# Supplementary material for: High Resolution Detection and Analysis of CpG Dinucleotides Methylation Using MBD-Seq Technology
Source: PLoS One. 2011 Jul 11;6(7):e22226. doi: 10.1371/journal.pone.0022226 (PMC3136941; doi:10.1371/journal.pone.0022226)
Supplement: Table S3 — PCR primer. (DOC) [file pone.0022226.s013.doc]

**Table S3. PCR primer**

| BiSearch Primer | Sequence 5'-3' |
| --- | --- |
| PLAU-F419 | GGGGTTTGAGGTAGTTTTAGGTAAGTT |
| PLAU-S875 | CTGCRAAAACAAATAAACCCTAACC |
|  |  |
| ARVCF-F49 | GGGGGGATTGGAGTTATTTTTA |
| ARVCF-R401 | AATACCTAACATATATATCTAAACAACCCTCTC |
|  |  |
| SBF1-F12 | GGGGGTGTATTTTGTATTTTGGT |
| SBF1-R383 | CTAACCATAACTTACCTAACCTCCTACTTAC |
|  |  |
| PFKL-F7 | GTGTTATTTGGGAAATTTTAGGTAGAAT |
| PFKL-R379 | ACATCCTAAAAATAACACCACAAAAA |
|  |  |
| HOXA11-F4+2 | GGTGTAATTTATGTTGGTTGGG |
| HOXA11-R4+3 | CTTCCCAAAACAAATCTATAAAAAAA |
|  |  |
| MC5R-F7+7 | TGTAGTTTATTGGTTATTGTAGTGGATAG |
| MC5R-R6-2+6 | CCAAAAAAAACATATATATATACAAAAACAC |
|  |  |
| ESPN-898F | TGGAAGGTAGGGTTTTTTGTAATTT |
| ESPN-1180R | CAAACAAACAAATTCATTCATCTACC |
|  |  |
| PLEKHG5-1146F | TGGTTTTTTTTTGTTAGGTAGAGAGG |
| PLEKHG5-1420R | ATATTCCCAAAACTTTACCAAAAAA |
|  |  |
| PIK3C3-883F | GATAGTTGAGAATAAGATGAGTATATGGTGT |
| PIK3C3-1198R | AACACTCCTACATAACCACCTTAAAAAA |
|  |  |
| KIAA0427-846F | AAAATTTTAGGAATTTAGGTTTTTAGTAGG |
| KIAA0427-1241R | CCTCACAAAACCCTCTTAATAAATAC |
|  |  |
| C18orf24-1187F | TTTATTTTTTTTTTTTTGGTTTGGG |
| C18orf24-1443R | AAACCATCCTTTAAACCTCTAAAAA |
