# Supplementary material for: High Resolution Detection and Analysis of CpG Dinucleotides Methylation Using MBD-Seq Technology
Source: PLoS One. 2011 Jul 11;6(7):e22226. doi: 10.1371/journal.pone.0022226 (PMC3136941; doi:10.1371/journal.pone.0022226)
Supplement: Text S1 — Supplementary methods. (PDF) [file pone.0022226.s014.pdf]

Supplementary Methods

Initial scan for target sites

Same methods as discussed in our lab's previous work [1] is applied.

A). Calculation of an average fragment length for a ChIP sample

The tag enriched region on both strands should be paired together at a target site and showing a bimodal pattern. In the first step, BALM sets a high stringent threshold (0.99995) and find ≥100 well-paired peaks, (if less than 100 pairs were found, the threshold is reduced and the process is repeated until more than 100 were found). Then, the program calculates the average fragment length at each of these high confidence target sites in Formula 1),

$$\hat{l} = \frac{\sum P_r}{n_r} - \frac{\sum P_f}{n_f} + 1 \qquad \textbf{1)}$$

Where P_f and P_r as the tag's position on forward and reverse strands respectively and n_f, n_r represent the number of reads on forward and reverse strands respectively.

To avoid excluding long fragments that have tags falling out of the enriched region, we extend the region by 200 bp on each side. The whole genome-wide average fragment length is computed $L = \hat{\hat{l}}$ and used to further shifting tags, search for possible target site etc.

B). Tag shifting

To estimate the fragment positions of the ChIP sample, we shift all reads towards the mid-point of the fragment by $L/2$ whereby each of these resulting points is considered as a representation of one fragment.

C). Determination of significant enrichment level thresholds

The genome is evenly divided into 50bp bins and the density of shifted tags for each bin is counted. A significant enrichment level threshold is calculated based on a percentile ranking method. After sorting the enrichment level for all bins, the threshold is taken at the percentile of the confidence level. By default, the confidence level is 0.995.

D). Definition of enriched regions and localization of the target sites

An enriched region is defined as a set of continuous bins that have an average enrichment level higher than the threshold. Initial scanning only allows gaps with one bin in length, since larger gaps might indicate that there are two closely located enriched regions. Then, we calculated the exact target site for that enriched region using Formula 2) with the assumption that the fragments are symmetrically distributed around a target site.

$$P_m = \frac{\sum(P_f - d) + \sum(P_r - d)}{n_t} \qquad \textbf{2)}$$

Where P_m denotes the target site (binding motif, modification site etc), n_t is the number of tags in the region, P_f and P_r as the tags' position on the forward and reverse strand respectively, d as the distance shifted (equals $L/2$).

The EM algorithm

Briefly, each tag x_i is treated as an independent observation, c_i determines which component of the mixture is x_i originated, k represent the kth component. Thus the components can be written as,

$$X_i \mid (C_i = k) \sim BALM(\delta_k, \sigma_k, \kappa_k)$$

Let τ_k denotes the portion of the kth component, thus, in a mixture model with m components,

$$\sum_{k=1}^{m} \tau_k = 1$$

σ and κ are treated as constants since these parameters are known and identical for all components. The unknown parameters to be estimated are,

$$\theta = (\tau, \delta)$$

The log likelihood function can be written as,

$$\ln L(\theta; x, c) = \ln \sum_{i=1}^{n} \sum_{k=1}^{m} \tau_k f(X_i; \delta_k) \quad \mathbf{3)}$$

where f is the probability density function of the bi-asymmetric-Laplace distribution, n is the number of observations.

Using Bayes' rule, the following formula could be derived to update the parameters for the next iteration [2],

$$\tau_k^{s+1} = \frac{1}{n} \sum_{i=1}^{n} S_{k,i}^{s} \quad \mathbf{4)}$$

$$\delta_k^{s+1} = \frac{\sum_{i=1}^{n} S_{k,i}^{s} x_i}{\sum_{i=1}^{n} S_{k,i}^{s}} \quad \mathbf{5)}$$

where *s* denotes the iteration step, $S_{k,i}^{s} = \frac{\tau_k^s p_k(x_i \mid \delta_k^s)}{\sum_{j=1}^{m} \tau_j^s p_j(x_i \mid \delta_j^s)}$ represents the conditional probability that x_i is from *k*th component at step *s*.

Data normalization and cross sample comparison

By default, robust linear regression [3, 4] is applied to normalize data with different sequencing depth since we assume that all experiments were performed under the same controllable conditions. A normalization factor is calculated by performing robust linear regression between tags count of 10kb bins of the two samples. Then the samples are adjusted to have comparable enrichment levels using the normalization factor. Fisher's exact test is applied to calculate a p-value for each enriched region as an indication of sample differences. Regions with a p-value less than 0.05 are defined as accepted regions otherwise are considered rejected regions. Both accepted and rejected regions are recorded as output.

FDR calculation

Same methods as discussed in our lab's previous work [1] is applied.

The procedure of synthetic data generation is described in the following section (**Generation of synthetic, simulated data**).

Let N_b denote the number of peaks detected in the synthetic data and N_t denote the total number of peaks detected from ChIP data. The FDR can be re-written as

$$FDR = \frac{N_b}{N_t} \times 100\% \quad \textbf{6)}$$

Generation of synthetic, simulated background data

We define a *signal-noise-ratio* (SNR) level as the following Formula, which is the basis for the monte-carlo simulation of data:

$$SNR = \frac{R_s}{R_o} \quad \textbf{7)}$$

Where R $(R_s = R_o)$ denotes the total number of reads, R_s denotes the number of reads that fall into peaks, and R_o denotes the number of reads that are not in any peak.

A). Simulated peaks (binding sites)

(a) Randomly generate *n* binding motifs (e.g. 10 nt in length) on the genome; (b) For each motif, generate a certain number of artificial fragments according to the ChIP sample; (c) Randomly define each fragment's length, varying from 100-300 nt; (d) Randomize each fragment's position around the motif; (e) One end of each fragment was randomly selected and its coordinates was recorded as one tag.

B). Randomly generate background noise reads

(a) After obtaining the coordinates of the reads in ***A)***, we randomly generate coordinates for background fragments throughout the genome. The number of fragments is equal to $R \times (1 - SNR)$ and the density of the noise fragments follows Poisson's distribution. (b) One end of each noise fragment was randomly selected and its coordinates was recorded as one tag.

C). Synthetic data

A synthetic data is defined as a dataset of a number of permutated tags within peaks in a corresponding sequencing data plus background noise tags. This dataset is used for the purpose of determining the FDR for each real data.

D). Simulated data

A simulated data is defined as a dataset of a pre-determined number of peaks plus background noise reads; this dataset is a combination of the tags generated in both ***A)*** and ***B)*** and is used for evaluation purposes.

BALM GUI

The GUI is implemented in Java, and has been tested with Windows, Linux and Mac OS X. To use it, specify parameters required, the locations of the input files, the BALM binary and the location to place its output, and click “Submit.” More detailed instructions are available in the provided manual.

References

1. Lan X, Bonneville R, Apostolos J, Wu W, Jin VX: **W-ChIPeaks: a comprehensive web application tool for processing ChIP-chip and ChIP-seq data**. *Bioinformatics* 2010.
2. Redner RA, Walker HF: **Mixture Densities, Maximum-Likelihood and the Em Algorithm**. *Siam Rev* 1984, **26**(2):195-237.
3. McKean JW: **Robust analysis of linear models**. *Stat Sci* 2004, **19**(4):562-570.
4. Taslim C, Wu J, Yan P, Singer G, Parvin J, Huang T, Lin S, Huang K: **Comparative study on ChIP-seq data: normalization and binding pattern characterization**. *Bioinformatics* 2009, **25**(18):2334-2340.
